# Health literacy in parents of children with Hirschsprung disease: a novel study

**DOI:** 10.1007/s00383-024-05917-4

**Published:** 2024-12-05

**Authors:** Signe Olsbø, Sara George Kiserud, Åsmund Hermansen, Marie Hamilton Larsen, Kristin Bjørnland

**Affiliations:** 1 Department of Gastrointestinal and Pediatric Surgery, Oslo University Hospital, Rikshopitalet, Postboks 4950 Nydalen, 0424 Oslo, Norway; 2 Institute of Clinical Medicine, University of Oslo, Oslo, Norway; 3 Department of Social Work, Child Welfare and Social Policy, Faculty of Social Sciences, OsloMet - Oslo Metropolitan University, Oslo, Norway; 4 Department of Behavioral Medicine, Institute of Basic Medical Science, Faculty of Medicine, University of Oslo, Oslo, Norway; 5 Lovisenberg Diaconal University College, Oslo, Norway

**Keywords:** Hirschsprung disease, Health literacy, Self-efficacy, e-health, Parents

## Abstract

**Purpose:**

To explore health literacy (HL) among parents of children with Hirschsprung disease (HD).

**Methods:**

Norwegian-speaking parents of children under 16 who underwent HD surgery at a tertiary center were surveyed using the Health Literacy Questionnaire-Parent, electronic Health Literacy Scale, General Self-efficacy Scale, and a study-specific questionnaire. Demographics were collected and ethical approval was obtained.

**Results:**

Among 132 parents (77 mothers) of 91 children (median age 8 years), high HL scores appeared in the domains “understanding health information” and “active engagement”, with lower scores in “provider support”, “health information appraisal”, and “social support”. Higher HL correlated with parents aged over 40 and higher education. Lower scores were seen with non-exclusive Norwegian use at home and not living with the child’s other parent. High electronic HL scores were common (mean 3.6, maximum score 5). 69% had high self-efficacy scores (score > 2, maximum score 4). Self-efficacy correlated strongly with higher HL scores.

**Conclusion:**

Parents of children with HD feel healthcare providers lack understanding of their child’s challenges, experience limited social support and struggle with interpreting health information. We suggest targeted HL interventions for young, lower-educated, non-cohabitating parents and those not primarily speaking the official language at home.

**Graphical abstract:**

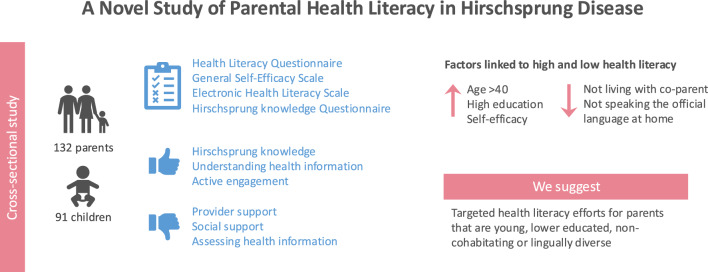

**Supplementary Information:**

The online version contains supplementary material available at 10.1007/s00383-024-05917-4.

## Introduction

Hirschsprung disease (HD) affects one in 5000 newborns with a male predominance of four to one. The condition involves the lack of ganglion cells in the myenteric and submucosal plexuses along a variable length of the distal gut, causing functional bowel obstruction [1]. Up to 30% of patients with HD have other comorbidities, Down syndrome being the most common involving around 10% of cases [2]. Although primary surgery for HD is generally successful, post-operative bowel dysfunction is common to varying degrees long term [3]. Bowel management in children with HD can be complex, involving medication, bowel evacuation routines, and special diets that require close parental control [4]. Parents coordinate care, communicate with daycare and schools, and are central in treatment decisions. They often need to cope with mental, physical, and social stress related to their child’s condition, which can negatively impact the daily life of both the child and the rest of the family [5].

Health literacy (HL) is the ability to access, comprehend, evaluate, and apply health-related information [6]. Enhanced HL is considered fundamental for future healthcare, enabling digitalization, home-based care, shared decision-making, and equity [7]. Parental HL encompasses a range of skills and competencies that allow parents to effectively navigate the healthcare system, understand medical instructions, communicate with healthcare providers (HCP), and make informed choices about their child’s health [8]. A recent systematic review on the relationship between parental HL and health outcomes for children with chronic diseases found a clear link between parental HL, health behavior and child health outcomes [9]. HL in parents of children with HD has not previously been studied. The aim of this study was, therefore, to explore parental HL in the context of HD and to investigate the possible effects of demographic factors and self-efficacy on parental HL.

## Methods

### Study design and recruitment

A cross-sectional study was conducted with parents of children under 16 years who had undergone HD surgery at Oslo University Hospital. The hospital is a tertiary referral center for pediatric surgery and treats around 80% of the country’s HD patients. The department participates in the European reference network ERNICA and offers multidisciplinary follow-up, including psychosocial support to families. We identified 137 patients who underwent HD surgery between 2007 and 2024 through patient records. Two patients had died and five had moved abroad, leaving 130 eligible participants. Primary caregivers able to answer the questionnaire in Norwegian were invited via mailed invitations or at the outpatient clinic by an independent person from October 2023 to May 2024. Participants could complete the form online or on paper and non-responders received a reminder after three weeks.

### Measures

#### Patient and parent characteristics

Clinical data such as diagnosis, surgeries, comorbidities and age at diagnosis were collected from records. Caregiver information, such as living situation, education, home language and work situation was collected via questionnaire.

#### Hirschsprung disease study-specific questionnaire

**Fig. 1:**
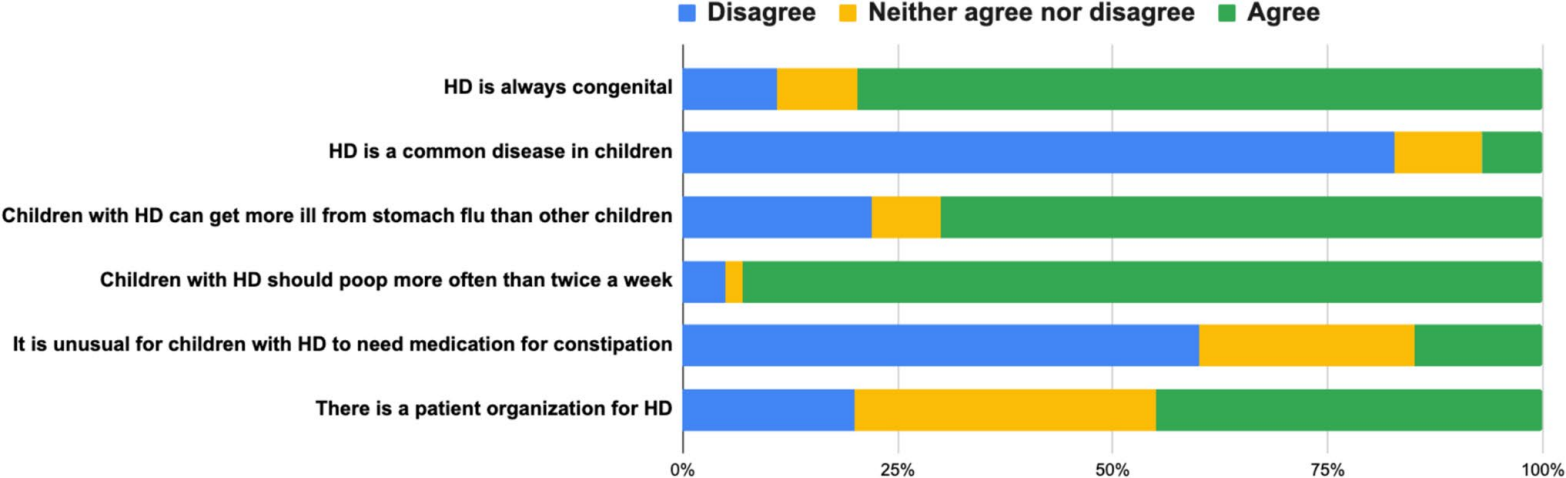
Parents’ general knowledge about Hirschsprung disease (HD)

A study-specific questionnaire on general knowledge about HD included 6 statements on basic facts and misconceptions (Fig. 1). This questionnaire was designed to get a general impression of the participants’ disease-specific knowledge about HD. We tested the questions with parents and colleagues and revised them locally. Parents used a 5-point Likert scale to indicate agreement. For analysis, “strongly agree” and “agree” were grouped as “agree”, and “strongly disagree” and “disagree” as “disagree”. A comment section was provided for additional remarks.

#### The health literacy questionnaire – parent (HLQ-p)

The HLQ is a generic, multidimensional instrument designed to assess an individual’s HL skills and abilities [10]. We used the parent-specific version of the HLQ (HLQ-p), which is a validated tool used to assess HL levels among parents in relation to the healthcare of their children [11]. It evaluates the ability of parents to access, understand and communicate health information related to their child’s healthcare. The questionnaire consists of nine domains relating to parental HL; (1) feeling that healthcare providers understand and support their child’s situation, (2) having sufficient information to manage their child’s health, (3) actively managing their child’s health, (4) social support for health, (5) appraisal of health information, (6) ability to actively engage with healthcare providers, (7) navigating the healthcare system, (8) ability to find good health information, and (9) understanding health information well enough to know what to do.

Responses for domains 1 to 5 are measured on a 4-point Likert scale ranging from “strongly disagree” to “strongly agree”, while domains 6 to 9 utilize a 5-point Likert scale assessing capability/difficulty ranging from “can’t do/always difficult” to “always easy”. A total score is not calculated for the nine HLQ-p scales. Instead, mean scale scores are calculated and interpreted separately (10). Scores > 2 on scales 1–5 signal a change from “disagree” to “agree”, and > 3 on scales 6–9 suggest a shift from “sometimes difficult” to “usually easy”, reflecting changes in HL without setting a fixed threshold for limitation. The HLQ-p has been validated in Norwegian with satisfactory results (11). The reliability of the HLQ-p was assessed using Cronbach’s alpha (Cronbach’s α 0.8–0.9).

#### The electronic health literacy scale (eHEALS)

The eHEALS assessed the participants’ perceived level of electronic HL (eHL), regarding finding, evaluating and utilizing online healthcare information [12]. The 8-question survey uses a 5-point Likert scale, with a higher score suggesting a higher level of eHL [12]. The tool has shown robust construct validity and reliability across various settings [13, 14]. Cronbach’s alpha was 0.9.

#### The general self-efficacy scale (GSES)

The GSES is a psychological assessment tool measuring the participants’ belief in their ability to handle challenges and accomplish goals [15]. Comprising 10 items, scores range from 10 to 40, with higher scores indicating higher self-perceived self-efficacy. For this study, scores were normalized to a 1 to 4 scale. The GSES has shown validity and reliability in studies on patients with different conditions [15]. Cronbach’s alpha was 0.9.

### Statistical analysis

Data analyses were performed using Stata 18.0. Initially, general characteristics were summarized using means and standard deviations. Independent *t*-test was used to compare differences between parent and child factors against HLQ-p domains. To assess the connections between HLQ-p, eHEALS, and various parental and child factors, bivariate correlation (Pearson’s R) was utilized. Next, a hierarchical linear multiple regression analysis in three steps was performed using the enter method; Step 1 including age, education and language; Step 2 living arrangements; and Step 3 involved adjustment for GSES score. The selection of variables included in the regression models was guided by the initial analyses. The associations are presented as standardized beta coefficients. Adjusted R^2^ explained variation in the associations.

A cluster variable for paired parents (88 pairs) ensured valid regression analysis, however, adjustments for clustering revealed no significant differences, therefore all parents (*n* = 132) were included. Significance was set at *p* < 0.05. The online form ensured no missing data for the HLQ-p, GSES and HD-questionnaire by making responses mandatory. We achieved a 98% completion rate for the optional eHEALS, which was deemed negligible for the analysis.

### Ethics

The project was ethically approved by the Regional Committee for Medical Ethics (REK; 402,216) and the Hospital’s Data Protection Officer (22/03367). All parents gave written consent. The children received age-appropriate information about the study.

## Results

### Cohort characteristics

Parents of 91/130 (70%) children completed the questionnaires. The median age of the children was 8 (0–15) years (Table 1). We received 132 parent responses, of which 79 (60%) were from mothers. Responses included 44 cases where both parents participated. In addition, one parent responded for 3 siblings, and another parent responded for 2 siblings. For the remaining 42 children, one parent responded to the child, providing a total number of 132 responses. Mean parental age was 39.8 years (SD 6.8), with no significant age difference between fathers and mothers (41.1 versus 38.9 years, *p* = 0.7). Most parents lived with the other parent of the child (79.5%), had higher education (52%), and worked full time (79%). 36% of the parents spoke another language or combined another language with Norwegian at home. Of the children, 75% were male, 74% had short segment HD and 28% had additional comorbidity, Down syndrome being the most common (13%).

**Table 1:** Demographics and clinical characteristics of children with Hirschsprung disease (*n* = 91) and their parents (*n* = 132)

Child characteristics	
*n*	91
Age in years, median (Min–Max)	8 (0–15)
Male sex, *n* (%)	68 (74.7%)
Length of aganglionosis, *n* (%)	
Rectosigmoid	67 (73.6%)
Descending/transverse colon	15 (16.4%)
Ascending colon	2 (2.2%)
Total colon	4 (4.4%)
Small intestine	3 (3.3%)
Age at diagnosis, *n* (%)	
Neonatal period	76 (83.5%)
1 month-1 year	8 (8.8%)
1 year-3 years	5 (5.5%)
> 3 years	2 (2.2%)
Age at pull-through, *n* (%)	
0–4 months	49 (53.8%)
4–12 months	17 (18.7%)
> 12 months	18 (19.8%)
No pull-through	7 (7.7%)
Additional medical history, *n* (%)	
Comorbidity*	22 (24.2%)
Down syndrome	12 (13.2%)
Current stoma, including appendicostomy	17 (18.7%)

Parent characteristics	
*n*	132
Age in years, mean ± SD	39.8 ± 6.8
Male sex, *n* (%)	53 (40.2%)
Living with child’s other parent, *n* (%)	105 (79.5%)
Level of education, *n* (%)	
Primary education ≤ 10 years	14 (10.6%)
Secondary education	50 (37.8%)
Higher education	68 (51.5%)
Work situation, *n* (%)	
Full-time work	101 (76.5%)
Part-time work	12 (9.1%)
Sick leave/unemployed/disability benefit	15 (11.4%)
Stay-at-home parent/student	4 (3.0%)
Language spoken at home, *n* (%)	
Norwegian only	84 (63.6%)
Norwegian and other language	34 (25.8%)
Other language only	14 (10.6%)

*Congenital heart disease, Down Syndrome, epilepsy, Mowat Wilson syndrome, Waardenburg syndrome

### General knowledge about Hirschsprung disease

The results from the HD study-specific questionnaire indicated that most parents had a good general knowledge about the congenital nature and rarity of HD (Fig. 1). They also recognized the necessity for regular bowel movements and acknowledged that children with HD can get more ill from stomach flu than other children. Awareness about the existence of a national patient association was limited.

### Health literacy, eHEALS and GSES scores

The average HLQ-p scores were above the critical low thresholds, with the highest scores in the domains “understanding health information well enough to know what to do” (domain 9) and “active engagement” (domain 6) (Table 2). The lowest scores were observed in the domains “feeling that HCP understands and supports my child’s situation” (domain 1), “appraisal of health information” (domain 5) and “social support” (domain 4). The parents generally demonstrated high eHL scores with 82% of the parents having a total score of > 3 points (maximum score 5), suggesting good ability to use electronic resources to manage their child’s health. For self-efficacy, the mean GSES score was 3.2 (SD 0.5, maximum score 4) with 69% of scoring high, defined as score > 2. GSES scores were comparable between mothers and fathers (mean score 3.2 versus 3.1, *p* = 0.5) (Table 2).

**Table 2:** Results from the Health Literacy Questionnaire—Parent (HLQ-p), the e-Health Literacy Scale (eHEALS) and the General Self-Efficacy Scale (GSES) questionnaires

Health literacy questionnaire-parent scores	*n* (%)	Mean (SD)	Min–Max	Cronbach α
1. Feel that healthcare providers understand/support my child’s situation*	132 (100)	2.6 (0.4)	1.2–3.2	0.8
2. Having sufficient information to manage my child’s health*	132 (100)	3.1 (0.6)	1.25–4	0.8
3. Actively managing my child’s health*	132 (100)	3.2 (0.5)	1.6–4	0.8
4. Experience social support for my child’s health*	132 (100)	2.9 (0.6)	1.4–4	0.8
5. Appraisal of health information*	132 (100)	2.7 (0.5)	1.4–4	0.8
6. Ability to actively engage with healthcare providers**	132 (100)	3.8 (0.7)	1.4–5	0.9
7. Navigating the healthcare system**	132 (100)	3.6 (0.7)	1.7–5	0.9
8. Ability to find good health information**	132 (100)	3.6 (0.6)	2–5	0.8
9. Understand health information well enough to know what to do**	132 (100)	3.9 (0.6)	1.8–5	0.8
Electronic Health Literacy Scale (eHEALS) Using technology to process health information 1–5 scale: strongly disagree, disagree, neutral, agree, strongly agree	128 (97)	3.6 (0.6)	1.1–5	0.9
General self-efficacy scale (GSES) Evaluates general sense of perceived self-efficacy 1–4 scale: Not at all true, hardly true, moderately true, extremely true	132 (100)	3.2 (0.5)	2–4	0.9

*1–4 scale: strongly disagree, disagree, agree, strongly agree

**1–5 scale: cannot do, very difficult, quite difficult, quite easy, very easy

### Factors influencing health literacy

Higher self-efficacy, living with the child’s other parent, and higher education correlated with higher scores in most HLQ-p domains (Table 3). Norwegian-only speakers at home and parents over 40 years also scored higher in certain domains. Parental sex and child-related factors such as the child’s age, time since diagnosis, length of aganglionosis, comorbidity or syndromes showed no correlation with HLQ-p scores and were therefore excluded from the multivariate regression analysis.

**Table 3:** Bivariate correlation coefficients (Pearson’s R), and significance (*) between the Health Literacy Questionnaire-Parent (HLQ-p) domains, electronic health literacy scale (eHEALS) and parental factors

	Age > 40	High education	Speaking only Norwegian at home	Living with co-parent	High GSES score
HLQ-p domains	r	r	r	r	r
1. Feel that healthcare providers understand/support my child’s situation	− 0.11	0.34**	0.14	0.34**	0.25*
2. Having sufficient information to manage my child’s health	0.21*	0.28**	0.16	0.18*	0.54**
3. Actively managing my child’s health	− 0.01	0.21*	0.07	0.17*	0.45**
4. Social support for health	0.03	0.26**	0.09	0.32**	0.41**
5. Appraisal of health information	− 0.01	0.23**	0.09	0.21*	0.39**
6. Ability to actively engage with healthcare providers	0.11	0.26**	0.23**	0.27*	0.48**
7. Navigating the healthcare system	0.13	0.37**	0.24**	0.14	0.56**
8. Ability to find good health information	0.16	0.33**	0.08	0.23*	0.49**
9.Understand health information well enough to know what to do	0.17*	0.43**	0.17*	0.27*	0.61**
Electronic health literacy scale (eHEALS)	− 0.09	0.07	0.08	0.06	0.16

A significance level of *p* < 0.05 was used

*Significant at > 0.01 level

**Significant at > 0.001 level

*r* bivariate correlations (Pearson’s R)

Bold values indicate statistically significant correlations (*p* <0.5) between health literacy domains and parental factors

In summary, the regression analysis revealed that parental age, language spoken at home, education and living arrangements significantly influenced HL scores (Table 4, supplement). Parents over 40 years scored higher in understanding health information and managing their child’s health (domains 2 and 9, St. β 0.2). Norwegian-only speakers scored higher in communication and healthcare system navigation (domains 6 and 7, St. β 0.3). Higher education correlated with higher scores in all domains (St. β 0.2 to 0.5).

When including living arrangements (Step 2), parents living together scored higher in most domains except in domain 7, navigation and 3, active management (St. β 0.2 to 0.5). Meanwhile, higher education remained significant for all domains except in domain 3, active management (St. β 0.2 to 0.4). Norwegian-only parents continued to score higher in communication and navigation (domains 6 and 7, St. β 0.3 to 0.4). When adding the GSES score (Step 3), higher self-efficacy correlated with higher scores across all HLQ-p domains (St. β 0.2 to 0.7). Cohabitating parents still scored higher in HCP support and communication (domains 1 and 6, St. β 0.3, 0.5), social support (domain 4, St. β 0.5), critical appraisal (domain 5, St. β 0.2) and finding and understanding health information (domain 8 and 9, St. β 0.3, 0.4). The final model explained 20–50% of the variance in the HLQ-p scales.

## Discussion

The main finding of this study exploring HL in parents of HD children is that the parents generally have good knowledge about the disease, but struggle with social and emotional aspects of caring for their child. A comprehensive study on HL in parents of children with HD has not been conducted previously, and our results offer several new insights.

Parents reported a lack of social support related to their child’s HD. We do not know the reasons for this but hypothesize that the stigma associated with defecation problems and the rarity of HD contribute to the sense of isolation [16]. Furthermore, Norway’s geography makes finding peers and support networks locally challenging. Besides, only half of the parents in this study were aware of the HD patient association, suggesting a possible source for peer support and shared experiences may be underutilized. Previous research has found similar issues among HD families [5, 17], with one study stressing parents’ lack of self-efficacy in seeking social support when caring for a child with HD [18]. Parents of children with anorectal malformation (ARM) experience similar psychosocial burdens [19], indicating a need for accessible support systems. Nevertheless, HCP should inform families about patient groups and support networks.

Parents generally perceived a lack of support and understanding from HCP about the child’s situation, which is surprising as our center offers HD families direct contact with their care team, including stoma nurses, and patients are routinely followed until age 18 with a transition consultation to prepare for adult healthcare systems. The reasons for this perception are not clear, but it is possible that specialized HD professionals unintentionally make parents feel overlooked in their efforts to normalize the condition and reduce over-medicalization. Additionally, some parents found interactions with general practitioners and emergency room staff challenging due to their unfamiliarity with HD, leading to difficulties in symptom interpretation and appropriate treatment. Effective family-centered care requires HCP to provide parents with appropriate information, discuss treatment options and value their preferences and concerns [20]. Improving these aspects is crucial in building trust and ensuring parents and their children feel supported and informed.

HD parents struggled with evaluating the quality and relevance of health information. This may contribute to their perception of being less capable of managing their child’s condition compared to parents of children with other chronic illnesses [5]. Since HD management is different for every child, parents need to adapt advice to their child´s specific needs, which requires critical HL skills [21]. If HCP acknowledge these challenges, they can give better support and help families feel confident.

### Sociodemographic factors influencing health literacy

Parental sex did not influence HL levels in this study. Some research suggests fathers are less engaged in health services than mothers [22, 23]. However, one study found higher communicative HL in fathers, although, they were also more educated than the mothers [24]. Our study is unique due to the high participation of fathers, possibly reflecting Norway’s emphasis on equal parental rights and social-gender equality. The similar HL levels in mothers and fathers may reflect mutual involvement in caring for a child with HD. Nevertheless, parental collaboration is crucial in alleviating the adverse impacts of chronic conditions on a child’s overall well-being [25].

We found that younger HD parents had lower HL, aligning with some, but not all studies on parental HL [23, 26]. Interestingly, time since HD diagnosis (a measure of experience) did not influence HL levels, suggesting that age, rather than experience, plays a role in enhancing HL. This may be due to parental experience and maturity and implies that young parents need extra support.

The finding that lower education predicts low HL is expected and consistent with global literature [9]. One study linked reduced HL to lower socioeconomic status, revealing barriers to care access and shared decision-making for those parents [27]. Academic education likely improves HL through accumulated knowledge and skills [7]. However, higher education does not guarantee high HL as many highly educated parents also had HL challenges.

Parents not living with the child’s other parent had more HL challenges. Research on social determinants for health in HD found that parental marital status affected a child’s risk of developing Hirschsprung-associated enterocolitis [28]. Similarly, unmarried maternal status has been linked to increased birth-related risks [29]. These findings underscore the need to consider family structure in HD management, suggesting targeted interventions for HL challenges in diverse family settings.

Language barriers and cultural disparities are known to complicate communication and HL and may even affect postoperative outcomes [30]. Immigrants and their Norwegian-born children make up roughly 20% of Norway’s population, and significant HL disparities exist among these communities [31]. Parents who spoke only Norwegian at home had better engagement with HCP and understanding of healthcare systems compared to those who also spoke another language. This suggests that even proficient Norwegian-speaking bilingual parents may have HL challenges related to language and that the use of interpreter services is crucial. Besides, excluding non-Norwegian-speaking parents likely skews our findings towards higher HL.

No child-related factors influenced parental HL. Since children with complex HD or comorbidity have more interactions with healthcare, we expected their parents to have increased HL. However, having a child with comorbidity, long-segment HD, permanent stoma or appendicostomy showed no link to improved HL. Research has not conclusively established a relationship between comorbidities and parental HL, and one study in fact linked comorbidity to *lower* HL [23]. Comorbidities may require parents to comprehend diverse information, potentially challenging their HL skills.

Our results point out self-efficacy as a strong predictor of parental HL, consistent with existing research in various pediatric patient groups [9, 23]. Enhancing self-efficacy through tailored interventions like education, mastery classes, and support networks could effectively improve HL in HD parents. Furthermore, parents demonstrated high levels of eHL, similar to findings among Swedish parents of children needing surgical care [32], suggesting eHL interventions could be effective. Electronic resources can provide accessible, tailored information, enabling informed decisions and active participation in their child’s care [33].

### Strengths and weaknesses

An important strength of the study is the authentic representation of the parent population. Oslo University Hospital treats about 80% of HD patients in Norway, and we evaluated HL in parents of 70% of these children. Families not included are those living in the northern part of Norway, typically with longer distances to the local hospital. Additionally, the study includes a substantial number of fathers and non-native speakers. Offering both online and paper surveys ensured diverse eHL levels. Another strength lies in the use of validated tools. Weaknesses involve the cross-sectional design lacking long-term follow-up, the relatively small population limiting advanced statistical analysis, lack of data on non-responders, and insufficient information on non-Norwegian-speaking parents’ HL. Lastly, the study-specific questionnaire has not undergone formal validation, so we cannot be certain that it accurately measures parents’ knowledge about HD.

## Conclusion

Parents of children with HD feel HCP lack understanding of their child’s challenges, experience limited social support and struggle with health information interpretation. HCP should address these barriers and offer targeted HL efforts to young, lower-educated, non-cohabitating parents, and to those who do not primarily speak the official language at home. Understanding these factors can guide tailored HL interventions to specific groups.

## Supplementary Information

Below is the link to the electronic supplementary material.

Supplementary file1 (DOCX 20 KB)

## Data Availability

No datasets were generated or analysed during the current study.
