# Supplementary material for: Health literacy in parents of children with Hirschsprung disease: a novel study
Source: Pediatr Surg Int. 2024 Dec 5;41(1):21. doi: 10.1007/s00383-024-05917-4 (PMC11618141; doi:10.1007/s00383-024-05917-4)
Supplement: Supplementary file 1 — Supplementary file1 (DOCX 20 KB) [file 383_2024_5917_MOESM1_ESM.docx]

**Table 4 (supplement):**

Steps 1 to 3 in the regression model demonstrating standardized beta coefficients (St. β), significance (*) and R^2^/adjusted R^2^ for the Health Literacy Questionnaire-Parent domains and the independent variables

| *Step 1* | **HPS**  St. β | **HSI**  St. β | **AMH**  St. β | **SS**  St. β | **CA**  St. β | **AE**  St. β | **NHS**  St. β | **FHI**  St. β | **UHI**  St. β | **eHEALS**  St. β |  |
| --- | --- | --- | --- | --- | --- | --- | --- | --- | --- | --- | --- |
| **Age** | -0.1 | **0.2*** | 0 | 0 | 0 | 0 | 0.2 | 0.2 | **0.2*** | -0.1 |  |
| **Language** | 0 | 0.1 | 0 | 0 | 0.1 | **0.3*** | **0.3*** | 0 | 0.1 | -0.1 |  |
| **Education** | **0.3*** | **0.2*** | **0.2*** | **0.3*** | **0.2*** | **0.2*** | **0.4*** | **0.4*** | **0.5*** | 0.1 |  |
| R^2^, adjusted R^2^ | *0.1, 0* | *0.1, 0.1* | *0.1, 0.1* | *0.1, 0* | *0.1, 0* | *0.1, 0* | *0.2, 0.2* | *0.1, 0.1* | *0.2, 0.2* | *0, 0* |  |
| *Step 2* | | | | | | | | | | | |
| **Age** | -0.1 | **0.2*** | 0.1 | 0 | 0 | 0.2 | 0.2 | 0.2 | 0.2 | -0.1 |  |
| **Language** | 0 | 0.2 | 0.1 | 0.1 | 0.1 | **0.4*** | **0.3*** | 0.1 | 0.2 | -0.1 |  |
| **Education** | **0.2*** | **0.2*** | 0.2 | **0.2*** | **0.2*** | **0.2*** | **0.4*** | **0.3*** | **0.4*** | 0.1 |  |
| **Cohabitation** | **0.3*** | **0.3*** | 0.2 | **0.5*** | **0.2*** | **0.5*** | 0.2 | **0.3*** | **0.4*** | 0.1 |  |
| R^2^, adjusted R^2^ | *0.2,0.2* | *0.2, 0.1* | *0.2, 0.1* | *0.1, 0.1* | *0.1, 0.1* | *0.2, 0.2* | *0.2, 0.1* | *0.2, 0.1* | *0.3, 0.2* | *0, 0* |  |
| *Step 3* | | | | | | | | | | | |
| **Age** | -0.1 | 0.2 | 0.1 | 0 | 0 | 0.1 | 0.1 | 0.1 | 0.1 | -0.1 |  |
| **Language** | 0 | 0 | 0 | 0 | 0 | **0.2*** | 0.2 | 0 | 0.1 | -0.1 |  |
| **Education** | **0.2*** | 0.1 | 0.1 | 0.1 | 0.1 | -0.1 | **0.3*** | **0.2*** | **0.3*** | 0.1 |  |
| **Cohabitation** | **0.3*** | **0.2*** | 0.2 | **0.5*** | **0.2*** | **0.5*** | 0.2 | **0.3*** | **0.4*** | 0 |  |
| **GSES** | **0.2*** | **0.6*** | **0.5*** | **0.5*** | **0.4*** | **0.7*** | **0.7*** | **0.6*** | **0.7*** | 0.2 |  |
| R^2^, adjusted R^2^ | *0.2, 0.2* | *0.5, 0.4* | *0.3, 0.3* | *0.3, 0.2* | *0.2, 0.2* | *0.3, 0.3* | *0.4, 0.4* | *0.3, 0.3* | *0.5, 0.5* | *0.1, 0* |  |

*Statistically significant p-value (p<0.05)

HPS: Feel that healthcare providers understand and support my child’s situation

HSI: Having sufficient information to manage my child’s health
AMH: Actively managing my child’s health
SS: Social support for health

CA: Appraisal of health information
AE: Ability to actively engage with healthcare providers

NHS: Navigating the healthcare system

FHI: Ability to find good health information

UHI: Understand health information well enough to know what to do
eHEALS: Electronic Health Literacy Scale
GSES: General Self Efficacy Scale
